# *In Vivo* and *In Situ* Approach to Study Islet Microcirculation: A Mini-Review

**DOI:** 10.3389/fendo.2021.602620

**Published:** 2021-05-10

**Authors:** Michael P. Dybala, Manami Hara

**Affiliations:** Department of Medicine, The University of Chicago, Chicago, IL, United States

**Keywords:** beta cell (β-Cell), islet, capillary, microcirculation, intravital 2-photon microscopy, 3D image analysis

## Abstract

The pancreas is regarded as consisting of two separate organ systems, the endocrine and exocrine pancreas. While treatment of a disease with either an endocrine or exocrine pathogenesis may affect the function of the entire pancreas, the pancreatic diseases have been treated by clinicians in different medical disciplines, including endocrinologists and gastroenterologists. Islet microcirculation has long been considered to be regulated independently from that of the exocrine pancreas. A new model proposes that pancreatic islet blood flow is integrated with the surrounding exocrine capillary network. This recent model may provide revived or contrasting hypotheses to test, since the pancreatic microcirculation has critical implications for the regulation of islet hormones as well as acinar pancreas functions. In this mini-review, practical applications of *in vivo* and *in situ* studies of islet microcirculation are described with a specific emphasis on large-scale data analysis to ensure sufficient sample size accounting for known islet heterogeneity. For *in vivo* small animal studies, intravital microscopy based on two-photon excitation microscopes is a powerful tool that enables capturing the flow direction and speed of individual fluorescent-labeled red blood cells. Complementarily, for structural analysis of blood vessels, the recent technical advancements of confocal microscopy and tissue clearing have enabled us to image the three-dimensional network structure in thick tissue slices.

## Introduction

In 1869, Paul Langerhans as a medical student first described the islet cells from rabbit pancreas as “small irregularly polygonal structures” that “gathered in rounded masses, 0.12-0.24 mm in diameter, distributed at regular intervals in the parenchyma”. Interestingly, Langerhans made these observations through the simple use of a primitive light microscope and multi-day fixation in Müller’s fluid (1). In the early 20^th^ century, islet morphology studies were performed using hematoxylin and eosin staining, which allowed researchers to appreciate islet morphology but not distinguish individual islet cell populations (2). Early descriptions of pancreatic beta- and alpha-cells were made using dye injections, since little was known about the functional differences of the endocrine cells (3). Lane was likely the first to differentiate alpha- and beta-cells in 1907, by observing morphological differences in islet cells when stained with aqueous-chrome-sublimate (beta-cells) versus alcohol-chrome-sublimate (alpha-cells). Approximately three decades later, Bloom described a method of differentiating beta-, alpha- and delta-cells using a single stain, the Mallory-Heidenhain azan trichrome technique, based on differential colors of cytoplasm and intracellular granules size and color (4). Elucidation of the function of islet endocrine cells allowed for implementation of a variety of histochemical methods to identify islet endocrine cells, including the Gömöri trichrome stain, pseudoisocyanin, and zinc and cobalt crystallization. Immunohistochemical (IHC) techniques were first described in 1942 as a method of detecting pneumococcal antigens using chemically-labeled antibodies (5). The use of IHC to identify pancreatic endocrine cells followed shortly after (6–8), though the IHC technique was not immediately widely adopted due to a lack of access to experimental tools such as antibody development and fluorescence microscopes (9). The development of IHC techniques allows for more specific and reproducible islet imaging as well as providing means of cell identification based on endocrine hormone production (e.g. insulin) rather than a chemical profile. Furthermore, fluorescent immunostaining for specific cellular products allows for simultaneous identification of multiple endocrine cell types within the same islet thus becoming a powerful tool for observing islet cell changes in both the healthy and pathological state. Parallel to the rapid development of unique staining techniques has been the rise of advanced microscopy, which allows for high resolution, multi-fluorescent, and even live-organism imaging (10).

Until recently, the pancreatic islet has mostly been studied using two-dimensional (2D) imaging even though the earliest trials of three-dimensional (3D) islet imaging took place as early as 1989 (11). 3D immunohistochemical microscopy has permitted visualization of the islet in its entire microenvironment and has provided new insight into islet architecture, such as the lack of a mantle consisting of non-beta-cells in rodent islets when observed in 3D (12). Such 3D techniques involve imaging of ~600-800μm thick slices of pancreatic tissue, whereas traditional 2D techniques use sections ~5µm thick, making the former more reasonable to visualize whole islets simultaneously since the vast majority of islets tend to be less than 200µm in diameter (13). Large-scale imaging of pancreatic tissue slices also permits adequate sampling of islets which are known to be heterogeneous in size, cellular arrangement, and cellular composition both within individuals and across individuals, particularly in humans. Limitations in interpretation of 2D imaging studies have been acknowledged, especially with consideration to the challenge posed by reconstructing 2D information into 3D and how such interpretation might have influenced how we would understand islet biology (14). Furthermore, the development of *in vivo* imaging has allowed us to visualize the dynamic pancreatic microenvironment in real-time. In this Mini Review, we will discuss the implementation of intravital islet imaging in mice and subsequent analysis of a large sample of data. Additionally, a procedural description of 3D imaging of thick pancreatic tissue slices and ensuing computer-assisted visualization and analysis will be presented. Overall, the rapid development of novel imaging techniques have allowed investigators to further their understanding of pancreatic and islet physiology. As such technological advances continue to change, though, precautions should be taken to understand potential limitations of contemporary techniques when interpreting experimental results.

## Conventional Methods Used for Studying Islet Microcirculation

The three previously discussed models of islet microcirculation, in which blood flows from non-beta-cells to beta-cells (model 1), beta-cells to non-beta-cells (model 2), or unidirectional *via* a gated portal system (model 3), were developed using distinct experimental methods (15). Proponents of model 1 used scanning electron microscopy to examine corrosion cast images of islet vasculature. Analysis of corrosion casts allowed for fine observation of microvascular details but failed to account for directionality of individual vessels, possibly contributing to the development of the insulo-acinar portal system hypothesis, in which efferent blood from the islets drains to the surrounding exocrine tissue. Furthermore, model 1 was built on studies of *in vivo* microscopy of ink-perfused islets under ultraviolet illumination. In these experiments, a fluorescent dye, either fluorescein isothiocyanate conjugated to bovine serum albumin or sulfoflavin S was injected into anesthetized rats. While *in vivo* imaging captures directionality of blood flow, the use of a dye makes it difficult to capture directionality of individual intra- and extra-islet blood flow. Model 2 was developed using corrosion casts and india-ink perfusion of rat islets followed by immunostained sections. Additionally, model 2 ascertained that afferent arterioles penetrated the islet core through gaps in the non-beta-cell mantle that help comprise the non-beta-cell mantle/beta-cell core structure that is necessary for the ordered perfusion of islets in this model. As we have previously shown, 3D reconstruction of a stack of 2D optical slices are necessary to appreciate the unique cytoarchitecture of the human islet. Analysis of immunostained 2D sections may lead to the perception of structural components, such as “composites of mantle-core cellular structures or subunits”, that are not seen when imaged in 3D (14). The researchers supporting model 3 utilized intravenous or intra-arterial infusions of fluorescent markers with subsequent examination under fluorescent light. They observed a “wave” of fluorescent markers move across islets under *in vivo* analysis, but a lack of highly specific tracking of individual cells may have contributed to interpretation of islet blood flow as afferent-to-efferent rather than recognizing the heterogeneous directionality of intra- and extra-islet pancreatic blood flow. Over a decade after the presentation of three models, Nyman and colleagues examined the proportion of these three models in mice using live imaging of islets with rhodamine-labeled dextran injection. They reported that all three models of blood flow were observed in mouse islets, with varying frequencies: model 2 > model 3 > model 1. Thus, this decades-long debate surrounding patterns of islet microcirculation remained unresolved, as the authors noted that the functional significance of finding multiple models of islet blood flow would require further study (16). It is noted that these previous models and the newly proposed one are mutually exclusive.

## Fluorescent Red Blood Cell (RBC) Labeling

**Figure 1:**
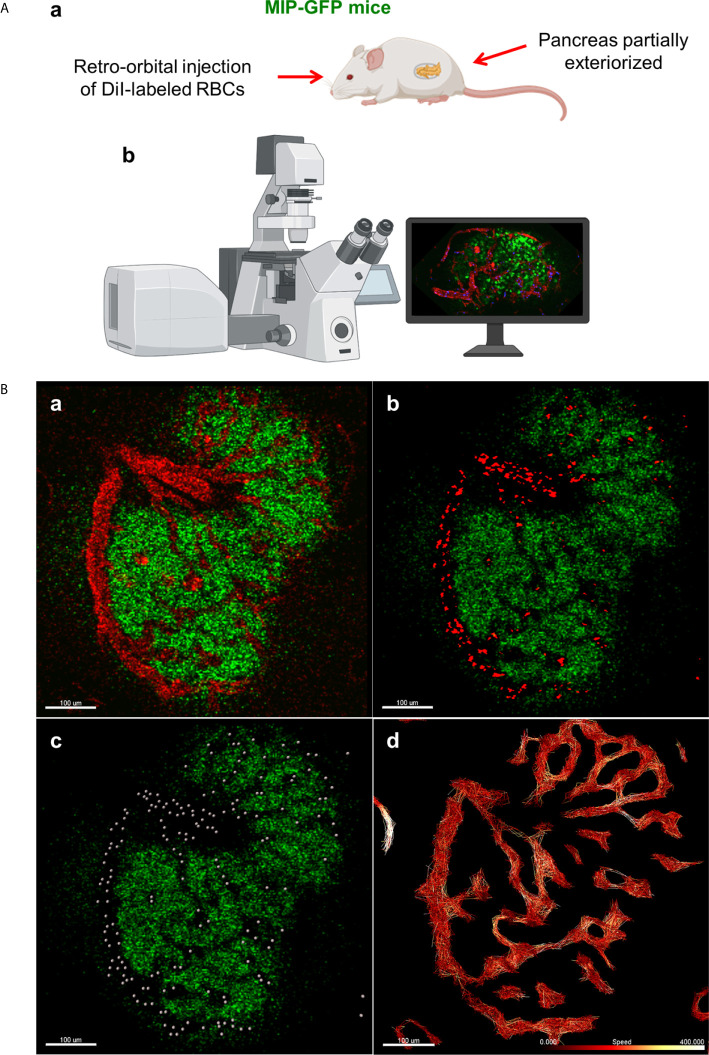
*In vivo* approach to study islet microcirculation in mice. **(A) a.** MIP-GFP mice are anesthetized with ketamine (100 mg/kg) (Fort Dodge AnimalHealth, Fort Dodge, IA) and xylazine (5 mg/kg) (Ben Venue Laboratories, Bedford, OH). After depilation, a pancreas is exteriorized. To visualize the blood flow, 10^8^ RBCs stained with DiI (Thermo Fisher Scientific, Waltham, MA) and/or tetramethylrhodamine (TMRD)-labeled dextran (2,000,000 MW) (Thermo Fisher Scientific) are injected intravenously in the mice just before imaging. **b.** Intravital microscopy is conducted using Leica TCS SP5 MP confocal microscope (Leica Microsystems, Mannheim, Germany). Images are recorded and analyzed using Leica LAS-AF as well as Fiji (http://imagej.net/Fiji). (Created with BioRender.com) **(B) a.** Fluorescent signal of dextran in mouse islet vasculature. **b.** Fluorescent signal of labeled individual RBCs in mouse islet vasculature. Scale bar: 100 µm. **c.** Computer-generated spheres representing tracked RBCs. **d.** Heatmap of RBC speed within the islet (fast to slow, white to red). Scale bar: 100 µm. (Adopted from 12).

In order to label and track individual RBCs in live mice, we used a similar method to one used to visualize fluorescent-labeled erythrocytes and leukocytes in mouse retinas (17). In transgenic mice in which beta cells expressed GFP under the control of the mouse insulin I promoter [MIP-GFP mice; (18)], the animals were anesthetized with ketamine (100mg/kg) and xylazine (5 mg/kg). Following anesthetization, 10^8^ fluorescent DiI-labeled RBCs were injected retro-orbitally, and the mouse pancreas was exteriorized following depilation. Intravital microscopy was conducted using the Leica TCS SP5 MP confocal microscope (**Figure 1A**). Advanced use of an abdominal imaging window for longitudinal studies of intravital imaging is described by Reissaus et al. (19).

## RBC Flow Analysis

Data from *in vivo* imaging are stored as .lif files and imported into Imaris image analysis software (Bitplane, Switzerland). Prior to performing RBC tracking analysis, the data must be stabilized, as perturbations from the mouse respirations can cause back-and-forth oscillations that will distort detected RBC flow patterns. In Imaris, such data can be modified for more accurate analysis using the drift correction tool, in which a stable reference point (e.g. the islet of interest) is used to modify the time series such that oscillations are reduced. Once drift has been corrected, RBC fluorescent signal is processed for easier flow analysis, using background subtraction and Gaussian blur features (**Figures 1B.a-b**). Using the Spots tool, individual RBCs can be tracked as individual objects, which provides detailed physical data, such as speed, velocity, acceleration, and displacement, among other measurements (**Figure 1B.c**). Specifically, when performing spot tracking analysis of mouse RBCs *in vivo*, we estimated the RBC diameter to be 6-8μm, which was confirmed with the measurement tool, and selected autoregressive motion tracking algorithm with a max distance between spots in subsequent frames of ~25µm. Imaris recommends that users only track spots in successive frames that can be distinguished with the human eye. That is, any uncertainty in the frame-to-frame identity of a single RBC, which may result from a low frame rate or extremely high RBC speeds, will make it difficult to precisely and reliably collect tracking data. Once RBC spot analysis has been completed, physical data can be collected for individual spots as well as for the paths on which the RBCs travel (length, straightness, average speed along track, maximum/minimum speed along track, etc.) (**Figure 1B.d**). Additionally, data can be filtered to only include RBC paths/tracks of a certain length, straightness, average speed, or physical location, as well as other variables.

## Optical Tissue Clearing

Successful 3D imaging requires optical tissue clearing, which allows for increased tissue transparency and the ability to image thick tissue slices with cellular resolution. Since the development of one of the most popular methods for tissue clearing, “CLARITY” (20), numerous other methods have been developed (21–23) to allow investigators to prepare tissues in a manner that aligns with their experimental goals. Many of these procedures are complex, requiring special devices and days to weeks of preparation. For example, CLARITY, which was coined from “Clear Lipid-exchanged Acrylamide-hybridized Rigid Imaging/Immunostaining/*In situ* hybridization-compatible Tissue-hYdrogel”, is based on hydrogel-tissue hybridization featuring lipid extraction by passive thermal diffusion and electrophoresis (20). Therefore, it requires a custom-designed chamber and continuous exchange of detergent (SDS solution) with the controlled gradual increase of the temperature. In the original protocol, relatively expensive solution FocusClear® (CelExplorer Labs, Hsinchu, Taiwan) is used for refractive index matching. This sophisticated method takes considerably long time from tissue preparation to imaging. We adapted the T3 method (24), which clears thick human pancreatic tissues in 1 day. The versatility of our method further stems from the use of primary antibodies conjugated with fluorescent dyes, which allows any combinations of primary antibodies regardless of their species-specific affinities. Our modified T3 method begins with washing antibody-incubated tissues slices for 30 minutes in PBS at 4°C. After washing, up to two tissue slices are sequentially incubated in 20, 50, 80, and 100% (v/v) solutions consisting of D-fructose in phosphate buffer with an additional 0.3% of α-thioglycerol for two hours each. Tissues are incubated using 10mL of solution in small glass vials wrapped in tin foil under gentle agitation at 36°C. Following incubation in the 100% D-fructose solution, cleared tissue samples can be stored at -20°C for several months based on our experience.

## Antibody Conjugate Preparation

Primary antibodies are first conjugated with secondary dyes overnight in a 1.5mL tube. The amount of primary antibody conjugated can vary depending on the volume needed for pancreatic tissue preparation but tends to fall in the range of 0.1-0.5mL. A 30:1 ratio of molar mass of antibody to mass of dye is used, which determines the required amount of dye for a given antibody volume (depending on the antibody’s concentration). Roughly 1-5μL of dye is required per 100μL of primary antibody. Following overnight conjugation *via* gentle agitation away from light at 4°C, the antibody conjugation is dialyzed in 1L of PBS at 4°C by injecting the conjugated antibody into a small dialysis cassette (Slide-A-Lyzer, Thermo-Fisher). The dialysis cassette should be spun on a low-speed setting throughout the dialysis process using a magnetic stir bar. The PBS solution is to be replaced with fresh solution three times: once in the afternoon of the same day as the start of dialysis, once the following morning, and once the following afternoon. Two days from the beginning of dialysis, the conjugate can be withdrawn and stored away from light in a tube at 4°C for up to six months.

## 3D Imaging

**Figure 2:**
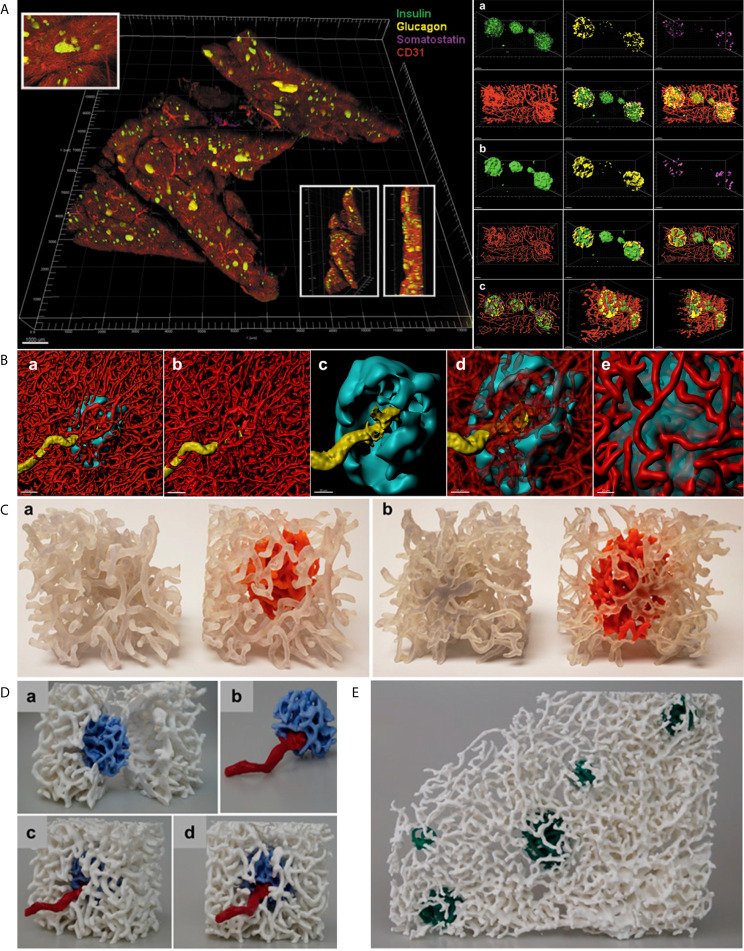
*In situ* approach to study the capillary network. **(A)** *Left*. Full scan of a 600 µm pancreatic slice immunostained for insulin (green), glucagon (yellow), somatostatin (magenta) and CD31 (red). Three insets for detailed islet resolution and displaying depth with different angles. Scale bar: 1000 µm. *Right*. **a.** Cluster of islets in the sample with individual fluorescent channel images and merged ones. **b.** Same cluster of islets converted to a computer-generated surface rendering with multiple angles displayed for 3D viewing. c. Coronal slice of all islets in the cluster and a sagittal slice of the leftmost islet visualized by use of a computer-generated clipping plane to reveal the intraislet blood vessels. Scale bars: all 50 µm. (Adopted from 25) **(B) a.** 3D-rendered view of a human islet integrated in the pancreatic capillary network. Islet (HPi1, a human pan-endocrine cell marker in cyan), blood vessels (CD31 in red), and an afferent arteriole (α-SMA in yellow). Scale bar: 50 µm. **b.** Blood vessels only, displaying the continuity of capillaries in the islet as well as exocrine tissue. **c.** The aforementioned feeding arteriole penetrating the center of the islet. Scale bar: 30 µm. **d.** Vasculature partially made transparent. **e.** Close-up view of the interface of capillaries entering and exiting the islet. Scale bar: 20 µm. **(C)** 3D prints of a human islet. **a.** Side-by-side comparison of the same region of pancreas with exocrine and endocrine (intraislet) capillaries printed the same color (left, clear) and the exocrine capillaries in clear and endocrine capillaries in orange (right). **b.** Opposite side. **(D)** A more expansive view of the endocrine/exocrine vascular network with feeding arteriole shown in **(B)** Blood vessels within the islet (light blue) are integrated with those in the exocrine tissue (white). A feeding arteriole (red) penetrates the center of the islet. **a.** A cross-sectional view of the islet capillary network within the exocrine tissue vasculature. **b.** Islet capillaries with a feeding arteriole. Note the markedly larger size of the feeding arteriole compared with endocrine blood vessels. **c.** Side view of the endocrine and exocrine vascular network. **d.** Front view of the endocrine and exocrine vascular network. **(E)** Human islet vascular network embedded in the exocrine vasculature. Blood vessels in five human islets (dark green) integrated with the pancreatic vascular network (white). (**B–D**, adopted from 12).

It is important to note that although we describe specific step-by-step methods for 3D modeling using a particular microscope and software, the same general principle of imaging and reassembling data points in 3D can be readily applied in other commonly-used and freely available software such as Fiji. Confocal 3D microscopy is performed using the Leica SP8 microscope in conjunction with Leica’s LASX imaging software (Wetzlar, Germany). Thick pancreatic tissue slices are examined under a 10x objective magnification. Specific features of the LASX software used to enhance imaging include increasing the bit depth from 8 (default) to 12, which captures finer details of the image. Additionally, the Super-Z mode is used to allow for visualization of up to 1.5mm of tissue, compared to the default range of 500μm. Multiple channel lasers and detectors are used (up to eight channels, where currently five are more optimal) in combinations of sequence bits such that the lasers in each sequence are on opposite ends of the emission spectra. Islets may be captured as part of a larger tile-scan, in which LASX stitches together a user-defined region of image tiles to capture an entire section of pancreatic tissue containing hundreds of islets. Additionally, single-tile images of a selected region may allow for higher quality analysis of an area containing several islets and their local environment. Importantly, adjusting certain parameters will markedly enhance or reduce the quality of image capture. First, reducing the z-step size in LASX will increase resolution of image details by capturing z-slices at smaller z-intervals, allowing for a more continuous structure when observed later in 3D (**Figure 2A**). We have imaged islets using z-step intervals as small as 0.5μm. Additionally, line averaging is a method used in which each X-line is scanned a user-defined number of times, and the result is averaged. Increasing the line averaging value is the most effective method for reducing noise in the resulting image. However, reducing z-step size and increasing the line averaging value will consequently result in longer scan times and larger file sizes. Generally, small z-step sizes and higher line averaging values can be used for imaging small regions of tissue, whereas large z-step sizes and lower line averaging values are required for whole-section tile-scans. For imaging small regions, we generally use z-step sizes of 1-2μm and line averaging values of 32-64. For larger whole-tissue sections, z-step sizes range from 6-10μm and line averaging values are generally 8-16.

## 3D Surface Rendering and 3D Prints

Files in “.lif” format can be loaded into Imaris for 3D surface rendering and analysis. Data from a tile-scan performed on the SP8 is imported into Imaris with all fluorescent channels available for analysis individually or simultaneously. For more specific analysis, such as analyzing a small region containing several islets and their associated vasculature, the Crop 3D function reduce the area of analysis. To process and clean the data, background signal/noise can be reduced by using the Thresholding-Baseline Subtraction function. Once background is reasonably reduced, the fluorescent signal for the structure of interest (islet, vasculature, beta-cells, alpha-cells, smooth muscle actin, etc.) can be transformed into a 3D structure using the Surfaces tool. First, the source channel must be specified for the structure of interest. Then, smoothing function within the Surfaces tool applies a Gaussian filter to the generated 3D surface, which can be useful in order to create smooth islet and vascular surfaces. In the next step, the user will manually select a threshold value to reasonably reduce background noise while including as much signal and structure of the desired object as possible. Once the algorithm completes, the user will have a set of surfaces that represent their fluorescent signal as well as accompanying data, which includes volume, surface area, sphericity, and x-y location, among others. Importantly, created surfaces can be used to “mask” another fluorescence within the same dataset, which allows for the capturing of fluorescent channels (and subsequent surface creation if desired) exclusively within or exterior to another surface. For instance, we have used the masking feature to measure the relative volume of capillaries within islets compared to the volume of each individual islet by masking the vasculature channel within a surface created for the islets (**Figure 2B**) (25). Furthermore, surfaces created in Imaris can be exported for 3D printing. The surface can be saved in Imaris as .vmrl file, which can then be imported into a free, open-source 3D mesh processing software (Meshlab) where the surface can be saved as a file compatible with 3D printing software (.ply and .stl, etc.) (**Figures 2C–E**).

## Discussion

Optical tissue clearing has been a key for successful 3D imaging of thick tissues. Among many reagents, we have chosen a sequential use of increasing concentrations of D-fructose supplemented with 0.3% α-thioglycerol to prevent browning (Maillard reaction) and autofluorescence (21). The refractive index of high-concentration D-fructose is higher than other water-based clearing reagents. Unlike sucrose, it does not cause tissue shrinkage. With this simple method, sufficient clearing of the pancreatic tissue is achieved in 1 day. Pre-conjugation of primary antibodies with an NHS ester-activated form of fluorescent dyes also accelerates the procedure, at the same time allowing for any combinations of primary antibodies without a concern of species-specific affinities. In terms of 3D imaging, a tile-scan of an entire tissue slice is useful for a large-scale unbiased analysis by capturing hundreds of islets at a time. Surface rendering is a powerful tool that includes many functions of Imaris such as panoramic views of islets and surrounding microenvironment as well as targeted transparency (e.g. intra-islet views). For *in vivo* studies of islet microcirculation in mice, direct RBC labeling with fluorescent dye and particle tracking functions of Imaris have enabled us to perform quantitative analyses.

The use of imaging modalities including both real-time *in vivo* microscopy of mouse tissues and 3D analysis of thick pancreatic tissue slices from various species enables investigators to study the endocrine and exocrine pancreas in an environment that more closely mimics the living organism. *In vivo* microscopy allows us to see movements within the tissue, but the recording of these motions occurs in one 2D plane, limiting our view of tissue depth. Whereas, the *in situ* approach provides an in-depth view of the 3D structure of the tissue, despite lacking a view of movements within the tissue. Together, these imaging modalities complement one another and help us integrate our observations by incorporating complex visual characteristics obtained from different modalities into a better understanding of islets and the pancreas. We further propose that there are still gaps in our knowledge of the evolutionary relationships among different species in terms of the distinct pancreas structures including islet architecture and vascular, neuronal and ductal networks. For example, intra-islet capillary is believed to be significantly less dense in humans than mice (26, 27). When islet size distribution is similar in these two species (13, 28), functional implications of this inverted intra-islet capillary density are unknown. Expanding the application of these methods to various species, particularly relevant to 3D imaging, such studies may shed light on some unanswered questions and further elucidate similarities and differences of the pancreatic structures among diverse species and the underlying evolutionary adaptations.

Along with the progression of methods from islet cell identification, as in the studies performed in the early 20^th^ century, to modern 3D rendering and real-time visualization, our interpretation of novel findings must also be adapted. We are looking at the same pancreas, but how we look at it has changed, which may provide us with a unique appreciation for pancreatic architecture and physiology that have not been recognized before.

Our understanding of the physiology and biology of the pancreas has been continually enhanced with the advancement of technology as described above. Interestingly, though, it is the human mind that could hamper such progress. Retrospectively, we may see something that does not exist, such as a non-beta-cell mantle of a rodent islet in 2D images that, we propose, may be due to our inherited visual perception, where Gestalt principles of continuity and closure are relevant (14). Inversely, we can fail to see something very new and unexpected that might have been right in front of us, especially when it challenges a gold standard that has dominated the field for a long time. In parallel with rapidly developing technology that allows us increasing resolution and maximal precision in our observations, it is important to remind ourselves to keep an open mind with good imagination.

## Author Contributions

MD and MH wrote, edited, and revised the manuscript. All authors contributed to the article and approved the submitted version.

## Conflict of Interest

The authors declare that the research was conducted in the absence of any commercial or financial relationships that could be construed as a potential conflict of interest.

The reviewer ST declared a shared affiliation with the authors, to the handling editor, at time of review.

## Abbreviations

GFP, green fluorescent protein; IHC, Immunohistochemistry; MIP, mouse insulin I promoter; RBC, red blood cell.
